# From Instability to Recovery: Mapping Youth Housing Trajectories with Life History Calendar

**DOI:** 10.1002/jcop.70102

**Published:** 2026-04-07

**Authors:** Thye Peng Ngo, Sara Semborski

**Affiliations:** ^1^ School of Nursing University of Michigan Ann Arbor Michigan USA; ^2^ Chapin Hall Chicago Illinois USA

**Keywords:** housing instability, identity and belonging, life course development, life history calendar, resilience and recovery, youth homelessness

## Abstract

Youth homelessness is widely studied, yet little research traces developmental housing pathways across time. This study used a life‐course lens to explore how youth navigate instability from childhood into emerging adulthood, and how connection, structure, and meaning‐making shape recovery. By mapping temporal movement, we identify turning points that shift trajectories toward stability. Eight youth (ages 21–26) enrolled in a cash‐transfer program participated in Life History Calendar interviews to reconstruct housing trajectories and contextual life events. Transcripts and calendars were analyzed using reflexive thematic analysis, incorporating within‐case mapping and cross‐case comparison. Eight interrelated themes traced developmental pathways into and out of homelessness. Early instability stemmed from caregiving rupture, institutional displacement, and accumulated adversity. Adolescence was marked by survival‐based autonomy, mistrust, and systems that supported and surveilled simultaneously. In emerging adulthood, youth rebuilt connections through peers, identity‐affirming community, and structured housing programs. Recovery was anchored in belonging, stability, and meaning‐making rather than individual effort alone. Youth homelessness unfolds as a developmental process shaped by intersecting trauma, inequity, and adaptive coping. Findings highlight the need for policies and interventions that reduce surveillance barriers and expand identity‐affirming, autonomy‐supportive housing models. Developmentally grounded support may strengthen long‐term stability and engagement.

## 1 Introduction

Youth homelessness remains a persistent public health and social inequity in the United States, affecting more than 4.2 million young people, or 1 in 10 young people ages 18–25, annually (Morton et al. 2018). The prevalence of homelessness is significantly higher among minoritized youth. More than one in four LGBTQ youth (28%) have experienced homelessness or housing instability at some point in their lives (The Trevor Project 2022). Racial inequities further widen these disparities, with homelessness occurring at twice the prevalence among White LGBTQ youth and nearly four times the prevalence among Black LGBTQ youth relative to their non‐LGBTQ White counterparts (Berger Gonzalez et al. 2021).

Homelessness is a profound social determinant of health, shaping exposure to physical, psychological, and structural stressors across the life course (Bowen et al. 2019; Saldua 2023). For youth, housing instability extends far beyond material deprivation; it disrupts access to safety, education, healthcare, and social connectedness, all foundational to healthy development (Hartswick Finch et al. 2025). When experienced during adolescence and emerging adulthood, homelessness interrupts identity formation, relational trust, and autonomy (Barker 2014; Magwood et al. 2019; Mayock 2023). These developmental disruptions are intensified by racialized poverty, family instability, and system involvement, which compound trauma risk and deepen health disparities (Bender et al. 2015; Edalati and Nicholls 2019; Embleton et al. 2016).

Homelessness is not an isolated event, but a cumulative developmental process shaped by exposures across the life course. Research consistently points to early predictors of youth homelessness, including family instability, poverty, substance use, mental illness, and child maltreatment, which intersect with structural inequities disproportionately affecting racial and ethnic minorities, immigrants, and LGBTQ+ youth (Giano et al. 2020). A meta‐analysis of 116 studies quantified these risks, showing that histories of foster care or incarceration tripled the likelihood of homelessness, with similar elevated odds among those who experienced physical abuse or drug use (Nilsson et al. 2019). These reviews highlight that pathways into and out of homelessness emerged through accumulated adversity. Yet, most studies treat these predictors as discrete correlates rather than trajectories of unfolding adversity. This study addresses this gap by using a life‐course lens to trace housing pathways and identify key transitions, turning points, and contextual factors shaping movement into and out of homelessness.

Using a life‐course developmental framework (Elder et al. 2003), this study examines how youth who experienced homelessness interpret their pathways across childhood, adolescence, and emerging adulthood. By tracing temporal patterns and contextual influences, we explore how youth describe key transitions and turning points shaping their housing trajectories, revealing processes of resilience, adaptation, and relational repair amid structural constraint. This approach centers lived experiences across time to inform developmental and policy perspectives on homelessness.

## 2 Methods

### 2.1 Study Design

We conducted a qualitative study using the life history calendar (LHC) method (Nelson 2019) to explore housing trajectories and related life events of youth experiencing homelessness. The LHC provided a visual and temporal framework to support recall, contextualize key life events, and co‐construct personal histories collaboratively with the researcher (Nelson 2019).

### 2.2 Participants and Recruitment

Participants were drawn from a parent study evaluating a cash‐transfer program for youth experiencing homelessness in San Francisco. Parent study eligibility required ages 18–24 and experiencing sheltered or unsheltered homelessness in the past 3 months (Semborski and Berger Gonzalez 2025), defined as lacking a fixed, regular, and adequate nighttime residence, including couch‐surfing, doubled‐up living, and other precarious situations (Morton et al. 2018; U.S. Department of Housing and Urban Development 2011).

This qualitative subsample included eight enrolled youth. Because LHC interviews occurred approximately 2 years after enrollment, several participants were older at interview than at baseline (Table 1 reports age at enrollment in the parent study). We used purposive maximum‐variation sampling to capture diversity across pathways linked to youth homelessness, including sexual and gender identity, foster care involvement, and justice system exposure (Almquist and Walker 2022; Dworsky et al. 2013; Morton et al. 2018). With a small sample, diversity was defined as variation across these domains rather than demographic representativeness (Table 1). Recruitment occurred through program staff referrals at a San Francisco youth‐serving organization.

**Table 1:** Participant Demographics (*N* = 8).

Participant	Age^a^	Gender identity	Sexual orientation	Race	Ethnicity	Housing status^b^	Housing stability level	Education^b^	Employment^b^	
P01	23	Transgender/non‐binary	Pansexual	–	Latine	Leased, independent housing	Stable	Not in school	Temporarily laid off	
P02	19	Cisgender woman	Heterosexual	Black/African American	–	Leased, independent housing	Stable	Not in school	Unemployed, seeking work
P03	23	Transgender/non‐binary	Unknown	White	–	Leased with Emergency Housing Voucher	Transitional	In a certificate program	Employed part‐time
P04	21	Transgender/non‐binary/woman	Gay or Lesbian	White	–	Leased, living with family & roommates	Stable but dependent	Not in school	Unemployed, seeking work
P05	22	Cisgender man	Heterosexual	AIAN	–	Transitional Living Program	Transitional	Not in school	Unemployed, seeking work
P06	24	Cisgender man	Heterosexual	Asian	–	Leased, independent, low‐income housing, at risk for eviction	At risk	In a community college	Unemployed, seeking work
P07	23	Transgender/genderqueer/woman	Gay or Lesbian	Multiracial (Black & AIAN)	–	Just moved into Permanent Supportive Housing	Moving from unstable, transitioning	In a career/technical program	Employed, seeking additional work
P08	19	Cisgender woman	Bisexual	PI	Latina	Transitional Living Program	Transitional	In a career/technical program	Unemployed, seeking work

a Age reflects age at parent study enrollment.

^b^ Housing status, education, and employment at the time of interview; AIAN=American Indian or Alaska Native; PI=Pacific Islander.

### 2.3 Data Collection

Participants completed a 40–60‐min one‐on‐one interview in which the researcher and participant co‐constructed an 18″ × 24″ LHC timeline plotting age (y‐axis) against major life events (x‐axis). The LHC visually mapped transitions across domains, such as housing, relationships, education, employment, health, and identity (Supporting Information).

A semi‐structured guide (Supporting Information) elicited reflections on turning points and contextual factors shaping each participant's housing trajectory from early childhood to young adulthood. Probes prompted discussion across personal (e.g., physical and mental health), interpersonal (e.g., family and peer relationships), social (e.g., education, employment, community), and structural (e.g., housing, justice, healthcare) domains. All interviews were audio‐recorded with consent and transcribed verbatim.

The LHC maps and transcripts were treated as complementary data sources: the calendars anchored temporal sequences, while the transcripts provided narrative depth and interpretation.

### 2.4 Data Analysis

We conducted a reflexive thematic analysis (Braun and Clarke 2021), informed by a life‐course perspective (Elder et al. 2003), to interpret patterns across Life History Calendars (LHCs) and interview transcripts. Coding combined deductive codes derived from the interview guide and life‐course constructs (e.g., housing transitions, relational context, institutional involvement, turning points) with inductive codes generated through iterative review (Fereday and Muir‐Cochrane 2006). Codes were organized into subthemes and then synthesized into overarching themes through memos, peer debriefing, and iterative refinement. Themes were selected for interpretive depth rather than prevalence (Supporting Information).

Analysis occurred in two stages. First, within‐case analysis integrated each participant's LHC and transcript to reconstruct their housing trajectories linking housing status with life events and turning points. Second, cross‐case analysis compared trajectories to identify shared and divergent patterns of instability and stabilization across developmental periods (Miles et al. 2014).

### 2.5 Reflexivity and Rigor

We approached the study from a constructivist and participatory perspective. The lead author, trained in qualitative and trauma‐informed interviewing, conducted all interviews with an emphasis on empathy, trust, and participant agency. The senior author contributed expertise in youth homelessness and policy contexts. Use of the LHC supported participant‐led narration. Rigor was strengthened through iterative coding, reflexive journaling, triangulation of LHC and transcript data, peer debriefing, and maintenance of an audit trail.

### 2.6 Ethical Considerations

The study was approved by the institutional review board. All participants provided informed consent, pseudonyms were used to protect confidentiality, and participants received $50 in appreciation for their time.

## 3 Results

We identified eight interrelated themes depicting youths' life‐course experiences of housing instability, relational loss, and recovery (Table 2). Though presented developmentally–from early disruption to later stability–these themes reflect overlapping, recursive processes, and ongoing negotiations of safety, belonging, and autonomy. Across narratives, youth moved in and out of stability, resistance, and resilience across their housing trajectories.

**Table 2:** Summary of Participant Trajectories Across Life‐Course Themes.

Participants	Theme 1: disrupted foundations	Theme 2: institutional control	Theme 3: adaptive survival	Theme 4: cyclical violence	Theme 5: navigating systemic	Theme 6: relational repair	Theme 7: stabilizing through structure	Theme 8: reframing pain into purpose
**P01**	Experienced early parental abuse	Hospitalized for mental health challenges	Began working at age 16 and relied on couch‐surfing	Experienced parental abuse and intimate partner violence	Faced discrimination from landlords	Developed peer support and belonging	Achieved sobriety and stability	Described this as the “first year of peace” and being housed
**P02**	Experienced maternal neglect and emotional deprivation	Multiple psychiatric holds and foster placements	Engaged in theft and street survival to meet basic needs	Experienced physical and sexual violence from peers	Reported racial discrimination	Built supportive peer relationships	Accessed therapy and TYI housing for stabilization	Described transformation into a “bulletproof girl” and a role model
**P03**	Lost housing following eviction and family instability		Relied on couch‐surfing for temporary shelter	Experienced conflict and aggression from family members	Faced housing discrimination and bureaucratic confusion	Developed support through partner and peers	Achieved financial stability through saving	Reported supporting others, including co‐signing a lease
**P04**	Experienced eviction and family instability	Hospitalized for mental health challenges	Relied on friends for housing and short‐term survival	Endured parental and partner violence		Developed community support through peer networks	Worked multiple jobs toward independence	Secured independent housing
**P05**	“Born into a gang neighborhood” and experienced parental neglect	Placed in juvenile detention, psychiatric facilities, and foster/group homes		Experienced intimate partner violence			Worked multiple jobs toward independence	
**P06**	Experienced family instability and multiple relocations	Spent time in juvenile detention and rehabilitation centers	Lived in a car and couch‐surfed during periods of instability	Separated from spouse and managed shared custody of daughter	Reported ambivalence toward housing assistance systems	Developed stability through TYI and other housing programs	Returned to college and maintained full‐time employment	Achieved housing independence and freedom from probation
**P07**	Experienced parental substance use and kinship adoption	Hospitalized for mental health challenges	Relied on couch‐surfing for housing					Achieved independent housing
**P08**	Born into homelessness; mother struggled with substance use	Lived in juvenile detention and group homes	Engaged in theft and couch‐surfing for survival	Experienced physical and mental abuse from sibling and partner	Encountered barriers to housing support		Re‐enrolled in college for a training program	Worked toward stable independent housing

*Note:* TYI = Trust Youth Initiative.

To situate themes developmentally, we reconstructed within‐individual housing trajectories linking housing status with key life events (Table 3). Instability began with early relational disruption and persisted through institutional placements and acute disruptions (e.g., violence, illness, eviction). Homelessness followed compounded relational, health, and economic events. Stability emerged when housing supports aligned with relational or motivational shifts, often through transitional programs acting as turning points. These trajectories provide the temporal and contextual foundation for the themes below.

**Table 3:** Within‐Individual Housing Trajectories and Linked Life Events.

P	Housing trajectory sequence	Key linked life events	Turning point to stability	Current housing
**P01**	Parental housing (0–14) → MH placements/stays with relatives (14–18) → Partner housing (18–20) → Couch‐surfing with parent (20–21) → Rented room (21–22) → Homelessness (22–23) → Transitional housing (23–24) → Shared housing (24–)	Parental divorce + abuse (14) → MH crises/placements; partner violence (18–20) → housing exit; injury + job loss (22) → rent loss → homelessness; substance use escalation (21–23); peer support → program entry ( ~ 23)	Transitional housing entry ( ~ 23) created a basic stability and recovery platform (“a place to go at night”)	Stable shared housing with peers since 24 (“first year of peace”)
**P02**	Maternal housing instability (0–11) → Displacement (11–12) → Foster/group placements (12–18) → Shelter (18–19) → House‐hopping/exploitative housing (19–20) → Transitional housing (20) → Hotel placement (20) → Supportive housing (20–)	Caregiver neglect → displacement (11–12); abuse disclosures → placements (12–18); institutional violence → instability; abusive relationship → exploitation (19–20); justice involvement → program referral ( ~ 20)	Supportive housing placement ( ~ 20–21) enabled safety and exit from survival strategies	Permanent supportive housing at 21; no longer engaged in survival sex or theft; describes desire to remain housed and safe
**P03**	Family friends (0–5) → Rental with parent (6–14) → Homelessness (14) → Rental with parent (14–21) → Relative housing (21–24) → Independent apartment (24–)	Caregiver substance use → instability; caregiver job loss/fire (14) → homelessness; household conflict/violence ( ~ 21) → exit; crime exposure ( ~ 22) → safety threat; employment income + voucher ( ~ 24) → housing access	Voucher + financial assistance ( ~ 24) enabled independent tenancy	Independent apartment since 24
**P04**	Parental housing (0–20) → MH placements (17–19) → Couch‐surfing (20–22) → Transitional housing ( ~ 21) → Rented room (22–)	Eating disorder + hospitalizations (17–19); caregiver conflict/violence (20) → housing exit; parental separation ( ~ 20) → housing loss; lack of income/credit (20–22) → couch‐surfing; education goal + program support ( ~ 22) → rental	Secured leased room for school ( ~ 22) (“my own space”)	Shared rented room since 22
**P05**	Foster/group placements (0–18) → Guardian housing (18–22) → Partner housing (22) → Hospital/recovery housing (22–23) → Migration for work (23) → Street homelessness (23) → Shelter/transitional housing (24–)	Chronic placement instability; early parenthood (15); violent assault + hospitalization (22) → housing disruption; migration attempts ( ~ 23); homelessness ( ~ 23); community/program connection ( ~ 24)	Youth shelter/transitional housing entry ( ~ 24) provided private space and planning support (“own personal space,” “privacy”)	Transitional housing since 24
**P06**	Extended family housing (0–15) → Juvenile facilities (15–18) → Adult incarceration (18–21) → Family housing (21) → Homelessness (21–23) → Subsidized housing (23–)	Family overcrowding/mobility (0–15); justice involvement (15–21); relationship separation + job loss (21–22) → homelessness; custody responsibilities ( ~ 22); housing assistance + waitlist ( ~ 23)	Subsidized housing placement ( ~ 23) enabled exit from homelessness (“finally housed”)	Affordable housing apartment (since 2023); currently housed with a pending eviction risk due to rent delinquency
**P07**	Adopted by aunt; housed in aunt's home (0–19) → abruptly kicked out after high‐school graduation (19) → prolonged couch‐surfing with cousins/sisters/friends (19–24) → own rented apartment (24–25)	Adoption after parental substance use; disability + income support; caregiver conflict → eviction (19); benefit loss ( ~ 22) → prolonged couch‐surfing; peer referral → housing program ( ~ 23–24)	Program engagement ( ~ 23–24) enabled the first lease	Permanent supportive housing since 24
**P08**	Shelter with caregiver (0–7) → Relative housing (7–13) → Relative/group placements (13–18) → Shelter alone (18–20) → Transitional living (20–)	Caregiver death (13) → relative care; abuse → juvenile placement (14–15); relationship conflict ( ~ 18); prolonged shelter stay (18–20); pregnancy during program (20)	Transitional living entry ( ~ 20) provided semi‐private housing and an education pathway	Transitional living program since 20

*Note:* P = Participant; MH = mental health.

### 3.1 Theme 1: Disrupted Foundations and Early Displacement

#### 3.1.1 Early Caregiving Rupture and Loss

Childhood was marked by caregiving disruption, as parental substance use, neglect, and unstable placements normalized instability. Participants described being “bounced house to house,” oscillating between kin, foster care, and institutions that fractured belonging. One youth shared, “I was always in foster care most of the time [since birth]” (P05). Others linked adoption to parental substance use (P07), including overdose‐related death (P08). Emotional deprivation compounded this instability: “I crave my mom's attention…I wasn't getting it anymore” (P02).

#### 3.1.2 Socio‐Structural Displacement and Racialized Barriers

Family instability intersected with economic precarity and structural inequities. One youth recalled, “My dad's job burned down, so we had to leave [our house]. We were homeless for a couple of months” (P03). Another described racialized housing discrimination: “[Rental owners] would hang up on my dad…because his name is Mexican.” (P01). A third reflected, “It's not my fault that my mom didn't want me…I didn't join a gang for no reason; I was just born into that area” (P05).

Across narratives, early instability and structural exclusion positioned mobility as survival rather than choice. These formative patterns of forced movement and adaptation shaped how youth later navigated safety, belonging, and stability.

### 3.2 Theme 2: Institutional Control and Betrayed Care

#### 3.2.1 Pathologizing Trauma as Deviance

In adolescence, trauma‐related behaviors were often reframed as deviance, placing youth in cycles of juvenile detention (P05 and P06), psychiatric holds (P02), and treatment programs (P01, P02, P03, P05, and P07) that emphasized control rather than care. One participant reflected, “I was in and out of juvenile detentions and treatment facilities…it wasted my whole middle‐school memories” (P05). Another shared, “I was only supposed to be gone for months, [but I] ended up being gone my whole childhood” (P02). Emotional distress was often treated as misbehavior rather than met with trauma‐informed support: “I was getting 5150 because [my mother] would kick me out…I would act crazy to get in the house” (P02).

#### 3.2.2 Educational Disruption and Institutional Fatigue

Institutional placements also disrupted schooling and peer relationships. Youth described losing formative years of education and connection: “I was in [juvenile treatment] for 2 years [and] wasted my whole friendship with a lot of people” (P05). Another shared, “I wasn't doing [well] in the last school, so they put me into a boarding school” (P08). Life history calendars revealed educational gaps that mirrored institutional stays, illustrating how “rehabilitative” systems disrupted continuity rather than restoring it.

Collectively, these experiences produced a sense of betrayed care. Institutions repeatedly misread trauma as misconduct, conditioning youth to expect control rather than support. Many left with heightened vigilance toward oversight and a strong preference for self‐reliance.

### 3.3 Theme 3: Adaptive Survival and Emotional Self‐Reliance

#### 3.3.1 Resource‐Driven Survivalism

With little dependable support, youth adopted pragmatic strategies to meet basic needs, including stealing, hustling, or couch surfing to secure food and shelter. “I used to steal anything to eat…that's how I would fend for myself,” one youth shared (P02). Others relied on federal government aid when health challenges made work difficult: “Having PTSD makes it hard to keep jobs” (P01). Another added, “I couldn't go to work because I was always sick from sleeping outside in the cold” (P02). Survival emerged as both resourceful and exhausting.

#### 3.3.2 Protective Autonomy and Withdrawal

Youth also cultivated protective autonomy by setting boundaries and limiting relationships to maintain control. “It was too much going house to house and other people's energies,” one explained (P07). Another set firm limits after achieving housing, “I put up boundaries with my mom—she's not allowed in my house at all” (P01). These strategies served as aself‐regulation, creating emotional distance in response to pervasive instability.

As youth entered adolescence and early adulthood, these defenses, while adaptive, sometimes reinforced isolation and mistrust. This tension shaped the next developmental shift, as youth navigated relationships that offered connection yet risked repeating past harms.

### 3.4 Theme 4: Cyclical Violence and Relational Fractures

#### 3.4.1 Re‐Enacting Trauma through Unsafe Relationships

Early mistrust resurfaced in adolescence and early adulthood through coercive or violent relationships, where partners and peers often replicated earlier dynamics of control and dependency. “It got really bad…I had to go to the police [because my ex‐partner] is a pedophile,” one youth shared (P01). Another described physical assault: “[My partner and I] got into a heated argument…and she stabbed me in my lung” (P05). These accounts show how unresolved trauma resurfaced in relationships that blurred care and harm.

#### 3.4.2 Gendered Vulnerability and Mistrust

Gender and sexuality shaped how youth experienced relational violence and rejection. Non‐binary and female‐identifying participants described intersecting gendered violence and transphobia: “The leaseholder's boyfriend was very anti‐trans…I couldn't say my pronouns.” (P01). Others encountered identity‐based family exclusion: “My dad wouldn't have been as big of a problem if I wasn't gay…he didn't want a gay daughter” (P04). These experiences deepened mistrust and reinforced withdrawal as a protection.

As youth moved away from harmful relationships, trust remained uncertain, making it difficult to distinguish supportive connections from those that echoed past harm. This tension set the stage for later themes focused on relational repair.

### 3.5 Theme 5: Navigating Systemic Contradictions

#### 3.5.1 Systems That Help and Hinder

Youth described welfare, housing, and probation systems as both supportive and discouraging–helping in moments of need while imposing requirements that felt demotivating. “The system is the ones that give you help, but at the same time, it makes you unmotivated because you're relying on the government,” one explained (P06). Another reflected, “People are still trying to help me, but people acted like they didn't want to help me…I'm just going with whatever comes” (P08). These accounts reveal how inconsistent support made systems difficult to rely on, even when intended to create stability.

#### 3.5.2 Bureaucratic Fatigue and Inequities

Housing and welfare navigation created ongoing fatigue through voucher rules, landlord stigma, and inequitable prioritization. “A lot of landlords don't want to pick Section 8 people because they think they'll ruin the house,” one explained (P03). Another noted gendered disparity: “[The housing system] doesn't prioritize men…they prioritize single moms or people with mental health disorders” (P06). Such encounters underscored how access to support was uneven and unpredictable.

These contradictions pushed youth toward resources with clearer expectations and more predictable pathways to stability, making a shift toward programs and communities that offered structure without the uncertainty embedded in bureaucratic systems.

### 3.6 Theme 6: Relational Repair and Conditional Support

#### 3.6.1 Healing through Meaningful Peer Relationships

In emerging adulthood, youth rebuilt trust through friendships and chosen family, in contrast to earlier instability. Peers offered accountability, guidance, and emotional safety: “My friend really helped me…I got up on my feet with that” (P01). Another described a friend “like a brother” who taught life lessons (P02). These relationships reintroduced care as mutual and reliable, allowing youth to practice vulnerability within safe boundaries.

#### 3.6.2 Shared Communities as Survival and Solidarity Networks

Belonging was also nurtured in collective spaces, such as punk communities, LGBTQ+ centers, and shared housing, where mutual aid and identity affirmation reinforced solidarity. “My housemates are super community‐oriented…we cook together, eat together,” one youth said (P01). Others accessed resources through identity‐based networks: “The LGBT Center helped me get the [housing] voucher” (P03). These communities offered emotional grounding and material support, functioning as informal safety nets when formal systems failed.

#### 3.6.3 Transitional Programs as Scaffolds of Stability

Structured housing programs, including the Trust Youth Initiative (TYI), provided stability through routine and material support. “[TYI] did help me…I have somewhere to go at night,” one participant explained (P01). Another reflected, “If it wasn't for them, I'd probably still be couch‐surfing” (P07). However, youth noted that such support remained conditional, shaped by program rules and expectations.

Through peers, community spaces, and transitional programs, youth gradually rebuilt belonging via safety, reciprocity, and guidance. These relational and structural supports laid the groundwork for the next developmental shift, as stability created autonomy and independence.

### 3.7 Theme 7: Stabilizing through Structure and Regulation

#### 3.7.1 Structured Independence and Financial Stability

Re‐engaging in school and work provided structure, accountability, and a pathway toward stability. Education offered direction, while employment was closely tied to securing and maintaining housing. “I want to work so I can get my housing,” one participant explained (P08). Others described financial gains beyond meeting basic needs: “[TYI] helped me save money…I co‐signed a lease [for my friend because] my credit score is really good” (P03). Financial momentum signaled growing responsibility, self‐discipline, and independence.

#### 3.7.2 Boundary‐Setting and Healthy Ties

Youth emphasized setting boundaries with family and partners to sustain emotional and housing stability. “I don't have to keep repeating this cycle within multiple people …even putting up boundaries with my mom” (P01). Some ended relationships to protect their well‐being: “We shouldn't be together because I don't want to feel depressed while I'm having a baby” (P08). Others maintained supportive ties: “There [are] people from placement that I still talk to… people who supported me through the bad times” (P02). These narratives show how youth balanced distance and connection to cultivate healthier relationships.

#### 3.7.3 Housing Structure and Stability as Mutual Stabilizers

Stable housing, emotional regulation, and relief from system oversight reinforced one another. “[With] structured housing…my nervous system is calming down, and I'm 6 months clean,” one youth shared (P01). Another described leaving probation as freeing: “I didn't have to check in…no probation officer [or] drug test” (P06). Many viewed housing programs as anchors: “If it wasn't for them, I'd probably still be [couch surfing]” (P07). These stabilizers enabled recovery, autonomy, and sustained independence. As stability grew through structure and routine, youth shifted from managing crises to imagining future goals, purpose, and contribution.

### 3.8 Theme 8: Reframing Pain Into Purpose and Contribution

#### 3.8.1 Transformation, Faith, and Reflection

As stability increased, youth reframed past adversity as growth and resilience. “A lot of [my mental health challenges] have gone away becoming an adult,” one noted (P01). Others turned to faith and therapy to sustain this growth: “I stopped stealing, stopped fighting…started turning to God…talking to my therapist” (P02). Reflection became both a marker and a mechanism of recovery, allowing youth to integrate hardship into a more coherent sense of self.

#### 3.8.2 Mentoring, Advocacy, and Reciprocal Care

For some, healing expanded into helping and advocating for others. One youth described becoming a “bulletproof girl” after trauma, offering support to peers: “People feel safe and comfortable to come talk to me” (P02). Others advocated for housing justice: “Big corporations [are] buying multiple properties, hiking up prices…nobody has a house anymore” (P01). These narratives show empowerment evolving into mentorship, solidarity, and a drive for justice.

#### 3.8.3 Integration and Future Orientation

Youth described a growing sense of peace, capability, and purpose as they integrated past experiences into an emerging adult identity. “[Age] 25 is my first year of peace,” one shared (P01), while another noted, “I'm standing on my own two feet…I don't ask for handouts” (P02). Others voiced concrete aspirations toward stable housing: “I'm doing what I could to be housed; I worked a few different jobs,” (P04) and “I'm working toward [my own apartment]” (P08). Youth reframed independence not as detachment, but as a balance of autonomy, connection, and purposeful forward movement.

## 4 Discussion

This study traced how young people experience and navigate housing instability across the life course, showing a developmental trajectory that begins with early caregiving disruption, moves through survival‐driven autonomy in adolescence, and shifts toward reconnection and meaning‐making in emerging adulthood. Homelessness emerged as a recursive process shaped by cumulative adversity, structural inequities, and adaptive strategies that offered short‐term protection but carried long‐term costs. Consistent with research conceptualizing youth homelessness as a developmental and ecological phenomenon (Milburn et al. 2024), our findings illustrate how instability builds over time, how youth negotiate safety and identity within constrained contexts, and how relational and structural supports enable recovery.

### 4.1 Early Childhood and Adolescence: How Early Disruption Accumulates Into Risk

Participants' early experiences of caregiving rupture, parental substance use, and repeated placements align with evidence that adverse childhood experiences heighten risk for later mental health challenges, educational disruption, and housing instability (Cutuli and Herbers 2014; Liu et al. 2021). By adolescence, trauma‐related behaviors were often interpreted as misconduct, leading schools, residential care, and juvenile justice systems to respond with exclusion or surveillance rather than support (Nichols and Malenfant 2023; Semanchin Jones et al. 2018). Such responses compound trauma, fuel institutional involvement, and contribute to instability; dually system‐involved youth face heightened risk for later criminalization (Narendorf et al. 2020). In our sample, these dynamics produced institutional fatigue and deepened self‐reliance, which accelerated adultification (Schmitz and Tyler 2016).

### 4.2 Late Adolescence: Survival Strategies as Adaptive and Costly

Survival strategies in late adolescence, including self‐reliance, emotional containment, and rigid boundaries, provided control but limited help‐seeking, intimacy, and trust, echoing Barker's (2014) concept of autonomy as both protective and isolating. Peer networks filled gaps left by absent adults and supported coping (Napoleon et al. 2024), but exposed youth to coercive and unsafe relationships (Petering et al. 2014, 2016). Gender and sexual identity shaped these risks. Women and LGBTQ+ youth, particularly youth of color, faced discrimination in services and heightened vulnerability (DiGuiseppi et al. 2022; Houghtaling et al. 2024). Structural systems also played an ambivalent role, as programs offered resources but imposed behavioral conditions and surveillance that undermined autonomy and trust (Dej 2020; Weisburd 2023).

### 4.3 Emerging Adulthood: Reconnection, Structure, and Meaning‐Making

Emerging adulthood provided opportunities for reconnection and stabilization. Youth emphasized the importance of consistent relationships, predictable structure, and supportive environments for rebuilding trust and pursuing self‐defined goals, aligning with developmental research on marginalized young adults (Sapiro and Ward 2020). Peer support, especially from individuals with lived experience, contributed to mental health and substance use recovery (Erangey et al. 2020; Gasior et al. 2018). While housing programs improved stability, housing alone was insufficient; youth‐friendly, low‐barrier spaces like drop‐in centers fostered deeper engagement, belonging, and health improvements (Curry et al. 2021; Rice et al. 2023; Slesnick et al. 2016). Meaning‐making through reflection, identity authenticity, community involvement, or advocacy emerged as a key mechanism of resilience (Hagler et al. 2025; Ketel and Abdoli 2025; Manoni‐Millar et al. 2025). These findings underscore that recovery is relational and structural, not simply a matter of individual grit.

### 4.4 Implications for Practice, Policy, and Research

Findings highlight that youth homelessness is a developmental and ecological process shaped by early adversity, institutional responses, and constrained opportunities for safety and belonging. Addressing these pathways requires coordinated reforms across systems that influence youth development from childhood through emerging adulthood, with implications for practice, policy, and research.

Practice must move beyond crisis response toward developmental support that reflects how youth rebuild stability over time. Upstream prevention through trauma‐responsive, family‐based interventions is essential (Wang et al. 2019; Wiewel and Hernandez 2022). Services should replace surveillance‐driven oversight with low‐barrier, strength‐based approaches that honor youth decision‐making and view self‐reliance and boundary‐setting as adaptive (Cumming et al. 2022; Dej 2016; Krabbenborg et al. 2017). Because relational repair and belonging supported recovery, programs should embed peer mentorship, community‐grounded care, and identity‐affirming spaces (Coolhart and Brown 2017; Curry et al. 2021). Streamlined eligibility processes can reduce system fatigue and improve access to needed resources and programs (Ecker et al. 2022). Finally, interventions must pair housing with opportunities to build agency, connection to community that allows young people to feel supported and independent, delivered through flexible, trauma‐informed, and developmentally aligned practices, with particular attention to the needs of young women, LGBTQ+ youth, and youth of color (de Pass et al. 2023; O'Shaughnessy and Michelle Greenwood 2020).

Current policies are designed for broad adult populations rather than the developmental needs of emerging adults, underscoring the importance of youth co‐design to promote stability and well‐being (Coffey 2023). Findings call for shifting from punitive, compliance‐focused approaches toward trust‐based, flexible, and developmentally appropriate supports. Housing reforms should expand low‐barrier entry and flexible income supports (Berger Gonzalez 2024; Homeless and Housing Resource Center 2024). Health policies must ensure access to identity‐affirming mental health care; models like CalAIM illustrate the potential of whole‐person care when implemented nationally (California Health Care Foundation 2021). Economic stability requires living‐wage employment and access to professional networks, alongside strong anti‐discrimination protections (National Network for Youth 2023; Romero et al. 2020). Education policies should expand postsecondary pathways, drawing from models offering foster youth free tuition and housing. Tax policy is another missed opportunity: uptake of California's foster youth tax credit remains low, and most young adults are excluded from the Earned Income Tax Credit (EITC) (Center on Budget and Policy Priorities 2023; John Burton Advocates for Youth 2025).

Research on youth homelessness remains fragmented, with limited longitudinal and mixed‐method studies tracing pathways from childhood into emerging adulthood or integrating structural, ecological, and developmental factors (Milburn et al. 2024). Our findings highlight the need for research that captures developmental inflection points and tests how relational repair, belonging, self‐efficacy, and community participation translate into long‐term stability. Comparative and intersectional research is especially needed for LGBTQ+ youth and youth of color, who face distinct structural and identity‐based barriers (Goodyear et al. 2024). Future work should also evaluate youth‐led and autonomy‐supportive models that empower rather than constrain development (Hagler et al. 2025).

### 4.5 Limitations

Several limitations warrant consideration. This study draws from a small qualitative subsample of youth participating in a San Francisco‐based cash transfer program, which limits generalizability but offers conceptual transferability to similar urban contexts. The strong local service infrastructure and program‐specific conditions may differ from settings with fewer resources or alternative policy environments. Although the LHC method enabled rich temporal reconstruction, retrospective accounts remain subject to recall bias. The visual co‐construction of timelines likely reduced this risk by anchoring memory around concrete events and transitions.

## 5 Conclusion

Youth homelessness reflects the cumulative impact of fragmented, punitive, and inequitable systems, and not individual failure. This study shows that stability emerges when young people are provided housing, flexible support, and affirming relationships that build autonomy rather than regulate it. Developmentally attuned, relational, and community‐rooted approaches have the potential to transform pathways from crisis toward connection, purpose, and leadership.

## Ethics Statement

The study was approved by the institutional review board at Chapin Hall.

## Conflicts of Interest

The authors declare no conflicts of interest.

## Supporting information

LHC_Supplemental_Materials_V2_03_20_2026.

## Data Availability

The data cannot be shared publicly due to the privacy of the participants in the study. The data will be shared on request to the corresponding author.
