# Supplementary material for: From Instability to Recovery: Mapping Youth Housing Trajectories with Life History Calendar
Source: J Community Psychol. 2026 Apr 7;54(3):e70102. doi: 10.1002/jcop.70102 (PMC13056351; doi:10.1002/jcop.70102)
Supplement: Supplementary file 1 — LHC_Supplemental_Materials_V2_03_20_2026. [file JCOP-54-0-s001.docx]

**Supplemental Materials**

**Life History Calendar Map (Example)**

**
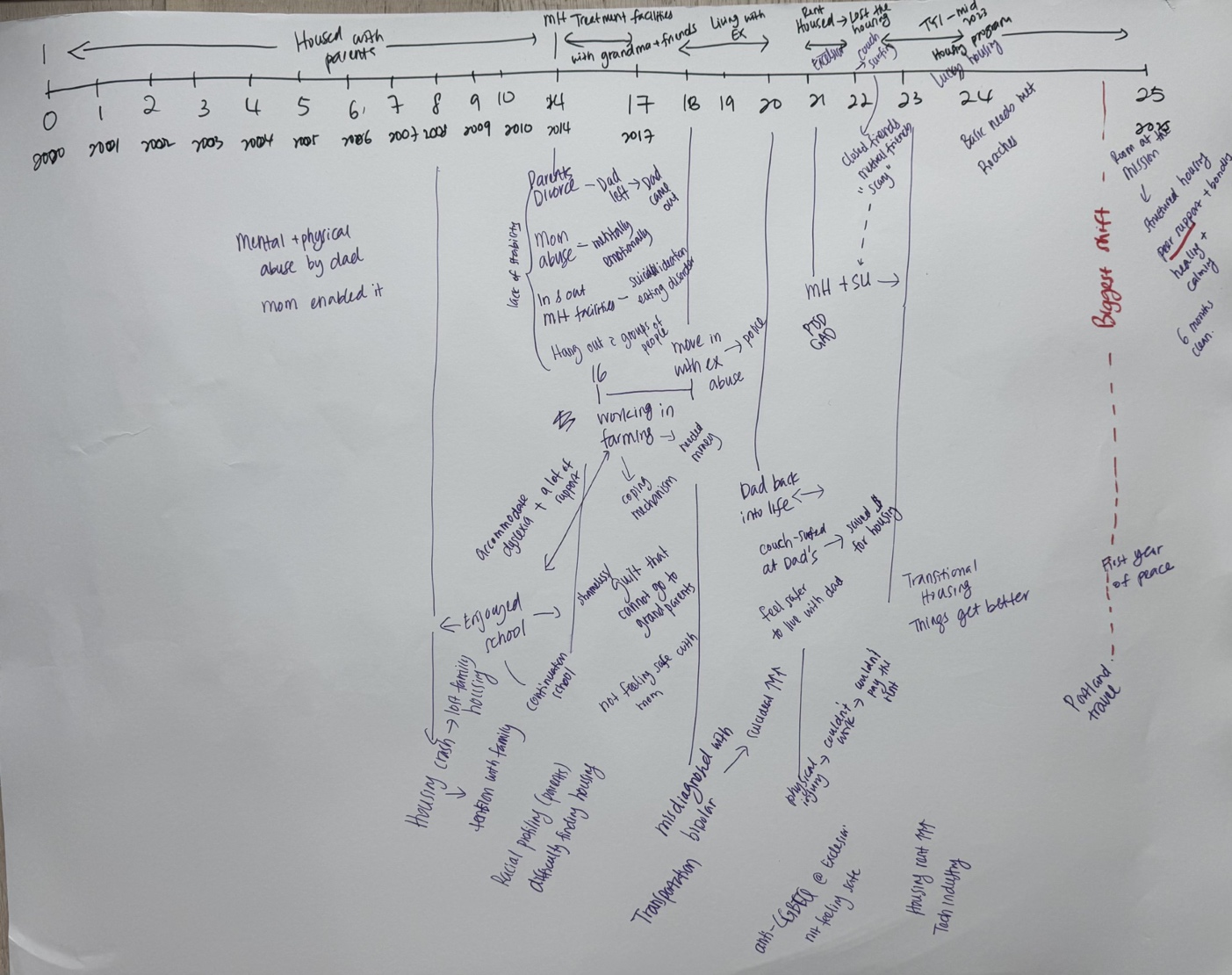
**

**Semi-Structured Interview Guide**

**Introduction & Rapport-Building**

“Thank you again for making time to meet today. Last time, we talked about your housing journey during your time in the Trust Youth Initiative. Today, we’ll take a step back and explore your broader life journey—experiences, relationships, and events that have shaped who you are and your path so far.”

“We’ll be using a tool called a Life History Calendar to help organize your story visually over time. We’ll co-create it together. The idea is to map out life events, transitions, and relationships across time, using time (like years or ages) on the top and life areas on the side. But we’ll decide together what those life areas are, based on what matters most to you.”

Additional Probes:

- How does this sound to you?
- Would you like to try it out together?
- What would make this conversation feel comfortable or authentic for you?

**Audio Recording Consent**

"Would it be okay if we audio record our conversation today? We can pause or stop the recording at any time."

**Co-Constructing the Matrix**

Instructions: Show the participant a blank matrix with time across the top (e.g., ages or years) and space for rows.

“Let’s think about different parts of life that have been important to you. We’ll write those down as the rows in the matrix. These might include things like places you’ve lived, people who mattered to you, school, work, or how you were feeling at different times. But we can add whatever feels most important to you.”

“We can start wherever makes sense for you. Some people begin at birth, others start now and work backward or jump around to the most meaningful parts.”

Additional Prompts:

- What are some of the big areas of your life you want to include?
- Are there any events or experiences that stand out?
- Who were the important people during different times in your life?
- What changed where you lived or how you felt about home?

[The goal is for the interviewer to be curious about the interconnected pieces of the young person’s life, temporally. We want to follow the young person’s lead. We do not need to discuss, nor should we attempt to discuss all topics, but some topics that we may be interested in hearing about include]:

- **Historical housing trajectory** (it may be easy to connect this to the previous conversation about their recent housing trajectory in Trust Youth Initiative).
- **Personal domains:** physical and mental health, substance use, identity formation.
- **Interpersonal domains:** family, romantic relationships, partners, peers
- **Social domains:** Education, employment, community belonging
- **Structural domains:** housing system, healthcare and other services, justice and immigration system

**During the co-constructing phase:**

- What was happening in this part of your life?
- How did this affect where you were living?
- Who was around you during this time?
- What changed or stayed the same?
- Probe if housing doesn’t come up: Would it be OK to talk a bit about where you were living during that time, and how that affected you?

**After completing the LHC matrix:**

- Looking at this timeline, do you notice any patterns or turning points?
- What has helped you survive and stay hopeful through these times?
- What do you think people misunderstand about young people who experience housing instability?

**Linking to the Trust Youth Initiative:**

- Based on what you shared, do you think any past experiences shaped how you engaged with the Trust Youth Initiative?

**Closing:**

- Is there anything else you want to share that we didn’t talk about?
- How are you feeling after this conversation?
- Thank you so much for sharing your story. Your voice and experience matter a lot.

**Within Case Analysis:**

| **ID** | **Themes** | **Subthemes** | **Supporting Codes** | **Analytic Level** | **Analytic Summary** | **Supporting Quotes** |
| --- | --- | --- | --- | --- | --- | --- |
| P01 | **Early Family Trauma and Instability** | Cycle of abuse and displacement | Abuse, Family, Divorce, Housing Loss | Personal / Interpersonal | Parental abuse and divorce triggered recurrent moves and housing loss, establishing both emotional and structural roots of instability. | “It was my parents' divorce, and my mom abusing me… It's more like mental abuse and physical abuse.” |
|  |  | Youth, identity, and poverty compounding risk | Abuse, Financial Need, Discrimination, Social Identity | Intersectional | Youth status, Latinx identity, and financial strain heightened exposure to violence and displacement. | “Just not having stable life to even go to school… As a teenager, there should be some stability to move through life, but it just wasn't there.” |
|  |  | Service deserts and transport barriers | Structural Barriers, Basic Needs | Community / Societal | Sparse mental‑health services and poor transit in rural areas deepened isolation and housing precarity. | “Where I lived… the bus lines… were horrible… there’s no programs for people like me… I had no one to go to.” |
|  | **Instability, Survival, and Coping** | Repeated mobility as trauma reenactment | Housing Transition, Couch Surfing, Mental Health | Personal / Interpersonal | Frequent moves between friends and relatives re‑enacted earlier instability and reinforced trauma cycles. | “I was just couch surfing between different multiple friends… some of them were really scary too.” |
|  |  | Work and withdrawal as survival mechanisms | Employment, Coping, Schooling | Personal | Work and periods of withdrawal provided temporary control amid persistent trauma. | “Farming was a coping mechanism… I was able to get a job through them.” |
|  |  | Abusive partners replicating early powerlessness | Intimate Partner, Safety, Abuse | Interpersonal | Coercive relationships reproduced earlier powerlessness and threats to safety. | “It got really bad… I had to go to the police… he’s a pedophile… I was gullible and easy to take advantage of.” |
|  |  | Housing–mental-health feedback loop | Mental Health, Injury, Housing Loss, Structural Barriers | Personal / Structural | Injury and mental‑health crises led to job loss and homelessness, sustaining instability. | “I burnt my foot… couldn’t work anymore… then I lost my place because I couldn’t pay for it.” |
|  | **Rebuilding Safety through Supports** | Family reconnection as fragile stabilization | Family Reconnection, Support System | Interpersonal | Reconnecting with father restored short‑term emotional and housing stability. | “My dad came back… I slept on his couch and then got my place… but it’s temporary.” |
|  |  | Peers and transitional housing as anchors of recovery | Peers, TYI/Housing Program, Support System | Interpersonal / Community | TYI and peer communities provided belonging, accountability, and daily structure critical for recovery. | “At the end, the [TYI] program did help me… I have somewhere to go at night… the simple things. Those really matter.” |
|  |  | Geographic and identity-based inequities in recovery | Structural Barriers, Social Identity | Intersectional | Access to affirming, resource-rich urban spaces enabled recovery unavailable in rural regions. | “In [San Francisco]… there are programs for people like me… on the coast side… they don’t have [programs] like here.” |
|  | **Recovery, Identity, and Stability** | Structured housing and sobriety as mutual stabilizers | Stability, Substance Use, TYI/Housing Program | Personal / Structural | Housing structure and sobriety reinforced one another, restoring emotional regulation and agency. | “Structured housing… it’s healing… my nervous system is calming down… I’m six months clean.” |
|  |  | Negotiating safety and belonging in gendered spaces | Social Identity, Safety, Renting | Intersectional | Nonbinary identity complicated safety in shared housing; belonging remained conditional. | “The leaseholder’s boyfriend… very anti‑trans… I couldn’t say my pronouns… I was scared.” |
|  |  | Autonomy through boundaries and healthy relationships | Independence, Support System, Peer Influence | Personal / Interpersonal | Setting limits with family and fostering healthy peer ties signaled psychological and spatial freedom. | “I can do my own thing… putting up boundaries with my mom. She’s not allowed in my house at all.” |
|  | **From Awareness to Advocacy** | Recognizing systemic and historical injustice | Structural Barriers, Discrimination, Social Identity | Community / Intersectional | Recognition of gentrification and racial profiling fueled advocacy and collective anger. | “I have deep anger… big corporations buying up multiple properties… hiking up the prices… nobody has a house anymore.” |
|  |  | Transitional programs as imperfect scaffolds | TYI/Housing Program, Support System, Employment | Structural | Programs offered immediate safety and community yet were bounded by geography and resources. | “The [TYI] program did help me… I have somewhere to go at night… the simple things.” |
|  |  | Transformation and forward momentum | Turning Point, Significant Shift, Mental Health | Personal / Interpersonal | Sobriety, community, and self‑advocacy redefined peace and stability as ongoing growth. | “[At age] 24… everything is starting to get normal… 25 is my first year of peace.” |
| P02 | **Early Neglect and Institutional Harm** | Maternal neglect and emotional deprivation | Abuse, Family, Basic Needs, Guilt/Shame | Personal / Interpersonal | Maternal neglect produced emotional deprivation, street survival, and feelings of unworthiness. | “I was left outside… ended up sleeping on a porch… I was a little girl outside begging for money.” |
|  |  | Exposure to violence and unsafe survival environments | Abuse, Safety, Peer Influence | Interpersonal | Sexual and physical violence during adolescence compounded neglect and reinforced hyper‑vigilance. | “I was raped at 12‑years‑old… held at gunpoint… because my mom kicked me out.” |
|  |  | Punitive placements over care | Housing Transition, Trauma | Community / Societal | Institutional placements prioritized control over care, amplifying instability and silencing. | “I was only supposed to be gone for months… I ended up being gone for my whole childhood.” |
|  | **Survival, Shame, and Adultification** | Premature survival strategies | Financial Need, Basic Needs, Employment, Couch Surfing | Personal / Interpersonal | Theft, hustling, and couch surfing forced premature adulthood. | “I used to steal anything to eat… that’s how I would fend for myself.” |
|  |  | Shame and anger as coping | Guilt/Shame, Mental Health, Coping | Personal | Persistent shame and anger reinforced low self‑worth and fighting as coping. | “I didn’t have self confidence… I was in a fighting stage… beating people up felt normal.” |
|  |  | Racialized surveillance and punishment | Structural Barriers, Discrimination, Housing Transition | Societal / Intersectional | Policing and prejudice deepened marginalization, perpetuating a survival–punishment cycle. | “It was a prejudice city… they treat Black people differently… I knew they were watching me.” |
|  | **Reframing Pain into Resilience** | Claiming a survivor identity | Mental Health, Trauma, Significant Shift or Event | Personal | Reflection and self‑advocacy transformed traumatic history into a survivor identity. | “I got the greatest progression… I don’t have to have a handout… I used to call it a bullyproof girl.” |
|  |  | Cultivating self‑regulation, faith, and gratitude | Turning Point, Coping, Support System | Personal / Interpersonal | Therapy and faith supported emotion regulation and intentional living. | “I stopped stealing, stopped fighting… started turning to God… talking to my therapist… people look up to me.” |
|  |  | Selective reconnection and trust rebuilding | Family Reconnection, Peers | Interpersonal | Rebuilt selective ties with family and peers who modeled care and accountability. | “There’s people from placement that I still talk to… people who were supporting me through the bad times.” |
|  |  | Becoming a role model and advocate | Independence, Support System, Turning Point | Personal / Interpersonal | Shifted from self‑protection to mentoring and advocacy. | “People feel like I’m that bulletproof girl… I’m standing on my own two feet.” |
| P03 | **Early Family Instability and Economic Insecurity** | Family displacement and job-related housing loss | Family, Housing Transition, Employment | Interpersonal / Structural | Father’s job loss and landlord eviction produced homelessness and reliance on extended networks. | “My dad's job burned down… we were homeless… the landlord gave us a month to move out.” |
|  |  | Parental substance use and role strain | Family, Substance Use | Interpersonal | Maternal substance use and uneven caregiving roles shaped guarded trust and self‑reliance. | “My mom has issues… a drug user… my dad thought he would be a better parent.” |
|  | **Housing Transitions and Safety-Driven Mobility** | Forced mobility due to safety threats | Safety, Trauma, Housing Transition | Personal / Interpersonal | Robbery and aggression motivated independent housing decisions linking safety with autonomy. | “There was a robbery… that’s why I left to rent apartment on my own… my brother’s boyfriend became aggressive.” |
|  |  | Navigating temporary living environments | Family, Support System, Safety | Interpersonal | Short stays with family provided relief but reintroduced conflict, reinforcing desire for autonomy. | “I stayed at my mom’s… then at her vacant house… only a couple months… then I left to rent on my own.” |
|  | **Program Engagement and Bureaucracy** | Community referrals and resource linking | TYI/Housing Program, Support System | Structural / Interpersonal | Community organizations (LGBT Center, TYI) facilitated access to housing resources and vouchers. | “The LGBT Center… helped me get the Section 8 voucher… TYI supported my housing progress.” |
|  |  | Financial literacy and saving behaviors | TYI Program, Financial Need, Coping | Personal / Interpersonal | TYI participation promoted saving habits and budgeting toward independence. | “[The TYI program] was really helpful… with saving money.” |
|  |  | Section 8 compliance anxiety | Structural Barriers, Discrimination, Housing Transition | Structural | Conflicting instructions and deadlines created fear of voucher loss and financial exposure. | “They told me I’d lose my voucher if I didn’t do an inspection… then said I didn’t have one due.” |
|  |  | Landlord stigma and price gouging | Structural Barriers | Structural / Intersectional | Stigma against Section 8 tenants and rent hikes reflected discrimination and affordability barriers. | “A lot of landlords don’t want to pick Section 8 people… places want to raise the price for Section 8 tenants.” |
|  | **Financial Responsibility and Emerging Independence** | Credit-building and relational trust | Independence, Peers | Personal / Interpersonal | Co‑signing and budgeting reflected confidence in managing risk and deepened accountability. | “I did cosign a lease for my best friend… my credit score is really good… I’m not really worried about it.” |
|  |  | Financial autonomy as a marker of adulthood | Renting | Personal / Interpersonal | Managing rent, credit, and savings signaled a shift from dependence to adult self‑sufficiency. | “[TYI] was really helpful with saving money… I left to rent an apartment on my own.” |
| P04 | **Early Family Trauma and Instability** | Family conflict, abuse, and forced exit | Abuse, Family, Safety, Housing Transition | Personal / Interpersonal | Escalating family conflict and parental abuse culminated in a violent incident forcing abrupt exit from the family home. | “He threw a ceramic mug at my head… I didn’t have anything on me other than my outfit and my wallet.” |
|  |  | Identity-based rejection and unsafe home environment | Social Identity, Discrimination, Family | Intersectional | Queer identity intensified family conflict, rendering the home unsafe and accelerating displacement. | “He didn’t want to have a gay daughter… that was a big point of contention.” |
|  |  | Parental divorce and emotional destabilization | Divorce, Family, Mental Health | Interpersonal | Parental divorce compounded emotional distress and destabilized family support during a critical developmental period. | “My parents got a divorce… she was also not safe in that situation.” |
|  | **Instability, Survival, and Coping** | Prolonged couch surfing and survival mobility | Couch Surfing, Housing Transition, Financial Need | Personal / Interpersonal | After leaving home, they cycled through friends’ homes, using mobility as a survival strategy amid limited resources. | “I didn’t live anywhere consistently… I was bouncing around.” |
|  |  | Informal care networks meeting basic needs | Basic Needs, Peers, Support System | Interpersonal / Community | Informal networks provided food, shelter, and emotional support, partially buffering material deprivation. | “I’ve stayed on friends’ couches… been fed by people when they were able to.” |
|  |  | Financial barriers to rehousing | Financial Need, Structural Barriers, Stability | Personal / Structural | Low income, lack of credit, and no permanent address constrained access to formal housing and prolonged instability. | “It’s hard to find housing if you have barely any income… no credit score… no permanent address.” |
|  | **Relational Trauma and Institutional Containment** | Abusive intimate partner relationships | Intimate Partner, Abuse, Safety | Interpersonal | Abusive relationships replicated earlier powerlessness, exposing them to severe injury and safety threats. | “They physically assaulted me… I couldn’t walk or talk for hours.” |
|  |  | Injury-driven hospitalization and educational disruption | Injury, Mental Health, Schooling | Personal / Structural | Severe injury and psychiatric hospitalization disrupted schooling and delayed housing and employment stability. | “I ended up being psychiatrically hospitalized… that was influential to my dropping out.” |
|  |  | Cyclical mental-health and housing instability | Mental Health, Housing Transition, Treatment Facilities | Personal / Structural | Mental-health crises and housing instability interacted, reinforcing cycles of disruption rather than stabilization. | “I was in and out of hospitalization… that fed into everything else.” |
|  | **Rebuilding Safety through Supports** | Transitional housing as temporary stabilization | TYI/Housing Program, Support System | Structural / Interpersonal | Transitional housing offered short-term relief and routine, while long-term permanence remained uncertain. | “That was good for a time… it’s nice to have a sense of stability.” |
|  |  | Maternal support and network bridging | Family Reconnection, Peers | Interpersonal | Maternal support bridged access to housing when peer networks collapsed after relationship loss. | “She helps connect me to people I could stay with… I don’t know what I would have done without her.” |
|  |  | Community and peer belonging through music | Interest, Peers, Social Identity | Interpersonal / Community | Local music and punk communities provided belonging, reciprocal aid, and emotional safety. | “If I was truly screwed, there are people that would help me.” |
|  | **Recovery, Identity, and Conditional Stability** | Employment as pathway to partial independence | Employment, Financial Need, Stability | Personal / Structural | Employment increased financial capacity and enabled rent payment, though stability remained precarious. | “It was definitely a good opportunity for me… before, I was quite broke… working in retail… I don’t think I would be able to afford my rent now if it weren’t for that.” |
|  |  | Identity safety shaping mobility and place | Social Identity, Safety | Intersectional | Queer identity informed geographic  and relational housing decisions | “Because I’m a visibly queer person… it doesn’t feel super wise to venture into unfriendly states.” |
|  |  | Stability defined through autonomy rather than permanence | Stability, Independence, Support System | Personal / Interpersonal | Stability was defined as autonomy, safety, and identity alignment, even when housing remained conditional. | “I’d rather not be somewhere miserable than be stable financially.” |
| P05 | **Disrupted Foundations and Displacement** | Early family instability and foster displacement | Family, Extended Family Housing, Couch Surfing, Housing Transition | Personal / Interpersonal | Foster care, unstable caregiving, and frequent moves normalized transience and weakened belonging. | “I was always in foster care… lived with relatives and people that are not relatives… always bounce house to house.” |
|  |  | Institutional control and loss of childhood | Treatment Facilities, Trauma, Schooling | Structural / Interpersonal | Juvenile detention and treatment replaced care with surveillance, costing schooling years and friendships. | “In and out of juvenile detentions and treatment facilities… it wasted my whole middle school memories.” |
|  |  | Violence in substitute homes | Intimate Partner, Trauma | Interpersonal / Personal | Assault within a romantic relationship perpetuated danger and mistrust. | “She stabbed me in my lung.” |
|  | **Street Networks and Survival Economies** | Peer‑based belonging and hood politics | Peer Influence, Structural Barriers, Substance Use | Community / Intersectional | Gang‑linked networks offered identity and protection while reinforcing exposure to crime and criminalization. | “It’s called hood politics… fights… somebody getting killed… rule was not to get caught.” |
|  |  | Scarcity‑driven risk normalization | Basic Needs, Substance Use | Personal / Structural | High costs and unmet needs made theft and substance use seem routine, reinforcing harm exposure. | “Why is food expensive… just taking care of myself… bringing weed to school and drinking.” |
|  | **Fragmented Caregiving and Attachments** | Substitute maternal figures and temporary stability | Extended Family Housing, Peers, Support System | Interpersonal | Care from non‑biological ‘mothers’ and friends’ families offered intermittent stability. | “My friend’s mom… took custody of me during that time.” |
|  |  | Selective family reconnection | Family Reconnection, Family | Interpersonal | Maintained limited ties (e.g., with sister) while protecting boundaries. | “I keep in contact with my sister.” |
|  | **Pursuing Independence amid Structural Instability** | Desire for autonomy and own place | Independence, Housing Transition | Personal / Structural | Attempts to live independently signaled maturation without sustained safety nets. | “I always wanted to have a place of my own… I lived by myself… for at least eight months.” |
|  |  | Employment as unstable pathway | Employment, Mental Health | Personal / Structural | Short‑term jobs provided income but triggered depression and quitting. | “Kitchen work… only for a month… started feeling depressed… decided to quit.” |
|  | **Moments of Growth and Insight** | Education as milestone | Schooling, Significant Event or Shift | Personal | High‑school graduation provided an anchor of achievement within disruption. | “I still graduated… that’s probably the only big event that was positive.” |
|  |  | Confronting trauma and early recovery | Substance Use, Support System, Treatment Facilities | Personal / Interpersonal | Treatment and recognition of suicidality fostered reflection and early recovery. | “My guardian told me to go to treatment… a sober community… get back on your feet.” |
| P06 | **Broken Foundations and Family Disruption** | Family instability and divorce | Family, Divorce, Stability | Personal / Interpersonal | A loving yet unstable family context and divorce perpetuated relationship and housing precarity. | “I value family… had my own family… went through infidelity… got a divorce.” |
|  |  | Extended kinship as diffuse support | Extended Family Housing, Family | Interpersonal | Large multigenerational networks offered belonging but limited financial safety. | “It’s not just my immediate family… like 30 of us… cousins, aunties… and their kids.” |
|  | **Structural Entrapment and Systems** | Control–aid paradox in systems | Structural Barriers, Housing Transition, Probation | Structural / Intersectional | Probation and housing supports sustained survival while reinforcing surveillance and dependency. | “You have to go through juvenile, adult system… homelessness just to get help… I was on paperwork until 2022.” |
|  |  | Perceived gender bias in access | Discrimination, Structural Barriers | Intersectional / Structural | Housing programs were seen as prioritizing single mothers or certain diagnoses over men. | “They prioritize single moms… they don’t prioritize men.” |
|  | **Unstable Housing and Economics** | Forced moves and near‑evictions | Structural Barriers, Financial Need | Structural | Rent delinquency and near‑evictions reflected chronic financial stress and vulnerability. | “Pending the eviction due to rent delinquency… we were damn near forced to leave.” |
|  |  | Reliance on family and vouchers | Family, Housing Transition | Interpersonal / Structural | Survival depended on extended family and hotel vouchers amid homelessness. | “I was homeless… lived in my car… hotel vouchers.” |
|  | **Reclaiming Direction** | Education as turning point | Schooling, Stability | Personal / Structural | Returning to college during homelessness marked a pivot toward long‑term stability. | “I started back my education… College of San Mateo.” |
|  |  | Housing programs scaffolding autonomy | TYI / Housing Program, Structural Barriers | Structural / Interpersonal | Larkin Street and TYI provided housing support while modeling responsibility and autonomy. | “[TYI] is a positive impact… preparing us to do it on our own.” |
| P07 | **Disrupted Beginnings and Displacement** | Parental substance use and caregiving loss | Family, Substance Use | Personal / Interpersonal | Parental addiction led to kin adoption and set the stage for cyclical displacement. | “I was adopted by my aunt because my mom’s on drugs… my dad was an alcoholic.” |
|  |  | Caregiver conflict and forced eviction | Family, Extended Family Housing | Interpersonal | Kin caregiving broke down, resulting in abrupt homelessness and relocation. | “My cousin drove me all the way out there [to my aunt's house], and all my stuff was outside.” |
|  | **Mobility and Fatigue** | Exhaustion from couch‑surfing | Couch Surfing, Extended Family Housing, Peers | Personal / Interpersonal | Rotating stays among relatives and friends produced emotional exhaustion and desire for solitude. | “Temporary. Cousins, sisters… It was exhausting… other people’s energy.” |
|  |  | Rotating kin/friend shelters | Peers, Extended Family Housing | Interpersonal | Survival relied on alternating help from relatives and friends. | “One year I stay with my cousin. The next year sister. Next year friend.” |
|  | **Institutional Interventions** | Behavioral struggles and schooling disruption | Schooling, Substance Use | Personal | Behavioral issues and expulsions. | “All I know was I was a bad child...Destructive, just all over the place.” |
|  |  | Mental-health treatment and control | Treatment Facilities | Structural / Personal | Hospitalization managed symptoms without addressing underlying trauma. | “I went to a mental hospital… they put me on medicine to calm down.” |
|  | **Survival and Navigation** | Meeting basic needs informally | Basic Needs, Financial Need | Personal / Structural | Without steady income, survival depended on theft or assistance from others. | “I probably just stole it… They gave me everything—food and stuff.” |
|  |  | Disability benefits as inconsistent safety net | Financial Need | Structural | Disability income intermittently stabilized finances but failed to secure independence. | “I already have my disability check… it stopped.” |
|  | **Toward Conditional Stability** | Program assistance enabling housing | Support System | Structural / Interpersonal | Youth housing programs provided short‑term stability and respite from family dependence. | “Programs… helped me out. If it wasn’t for them, I’d probably still be [couch surfing].” |
|  |  | Desire for autonomy and distance | Couch Surfing, Peers | Personal | Sought privacy and autonomy while maintaining cordial ties. | “I just don’t like being around other people… our relationship is really good.” |
| P08 | **Early Instability and Maternal Loss** | Homeless beginnings and maternal substance use | Family, Substance Use | Personal / Interpersonal | Early homelessness and maternal addiction seeded insecurity and abandonment. | “When I was born my mom was homeless… she was in a shelter… [she] passed away from alcohol poisoning.” |
|  |  | Parental neglect and loyalty conflicts | Family | Interpersonal | Lack of maternal protection during abuse generated mistrust within family ties. | “My brother physically abused me… [my mother] was picking his side.” |
|  | **Violence and Unsafe Relationships** | Abuse in family and partners | Abuse, Safety, Intimate Partner | Interpersonal | Physical and emotional harm from relatives and partners reinforced trauma and eroded safety. | “I don’t feel safe living with my brother… [my partner] insults me… hurtful things about the baby.” |
|  |  | Boundary‑setting for emotional safety | Intimate Partner, Turning Point | Personal | Pregnancy and reflection prompted separation from harmful partners to protect mental health. | “We shouldn’t be together… I don’t want to feel depressed while I’m having a baby.” |
|  | **Navigating Systems for Stability** | Transitional housing and partial safety | Housing Transition, Structural Barriers | Structural / Personal | Shelters and shared housing offered temporary security but limited autonomy and clarity. | “I felt safe with my aunt and grandma… but I wish it had been my own apartment.” |
|  |  | Barriers to support and system fatigue | Structural Barriers, Support System | Structural | Repeated attempts to access housing met indifference, fueling frustration and confusion. | “People act like they don’t want to help me… I go around different programs to ask.” |
|  | **Resilience through Work and Education** | Employment and skill development | Employment, Schooling | Personal | Sustained work and schooling reflected agency and drive for self‑sufficiency. | “At 19 I worked as a counselor… I started the medical assistant program in October 2024.” |
|  |  | Educational persistence amid disruption | Schooling, Turning Point | Personal | Boarding school provided structure and motivation to value education for stability. | “I started to take school more serious [when I was in boarding school].” |
|  | **Evolving Agency and Coping** | Emotional regulation via companion animal | Support System, Mental Health | Personal / Interpersonal | Attachment to a support animal provided comfort and responsibility as early self‑care. | “I had a dog support animal… I’ll be missing her… I can’t have her in the program.” |
|  |  | Shifting from dependence to autonomy | Turning Point, Intimate Partner | Personal / Interpersonal | Insight into relational dependence spurred a move toward independence and self‑definition. | “Relationships slow me down… I shouldn't always depend on people all the time.” |

**Cross-Case Analysis:**

| **Theme** | **Subtheme** | **Analytic Level** | **Analytic Summary** | **Integrative Summary** | **Core Process / Mechanism** | **Participants** | **Exemplar Quotes** |
| --- | --- | --- | --- | --- | --- | --- | --- |
| **Disrupted Foundation and Early Displacement** | Early caregiving rupture and loss | Personal / Interpersonal | Parental substance use, neglect, and unstable caregiving fractured early attachments and normalized emotional and spatial instability. | Childhood instability emerged through disrupted caregiving and structural displacement. Early attachment ruptures set the groundwork for mobility as survival—a learned adaptation that persisted into adolescence and shaped mistrust of care systems. | Attachment rupture and loss | P01, P02, P05, P07, P08 | P05: "When I was born, I was always in foster care most of the time." P02: "I crave my mom’s attention… I wasn’t getting it anymore." P07: "I was adopted by my aunt because my mom’s on drugs… my dad was an alcoholic." |
|  | Socio-structural displacement and racialized barriers | Interpersonal / Structural | Economic instability, discrimination, and neighborhood violence compounded instability, pushing families into repeated displacement. |  | Structural insecurity and racialized exclusion | P01, P03, P05 | P01: "[Rental owners] would hang up on my dad… because his name is Mexican." P03: "My dad’s job burned down so we had to leave our house.” P05: "I didn’t join a gang for no reason; I was just born into that area." |
| **Institutional Control and Betrayed Care** | Pathologizing trauma as deviance | Structural / Interpersonal | Youth whose trauma-related distress was reframed as behavioral deviance were controlled, medicated, or confined instead of supported. | Adolescence marked a stage where care systems transformed trauma into pathology. Institutional settings across education, juvenile justice, and treatment reframed distress as disorder and enforced compliance over care, eroding trust and reinforcing exclusion. | Institutional pathologization and coercive control | P02, P05, P07 | P07: "They put me on medicine to calm down… getting kicked out of schools." P02: "I was only supposed to be gone for months… ended up being gone for my whole childhood." P05: "In and out of juvenile detentions and treatment facilities… it wasted my whole middle-school memories." |
|  | Educational disruption and institutional fatigue | Structural / Community | Repeated placements and expulsions fractured schooling, social ties, and identity formation, making education collateral damage of institutional punishment. |  | Punitive displacement and loss of developmental continuity | P01, P02, P05, P07, P08 | P05: “I was in [juvenile treatment] for like two years… wasted my whole friendship with a lot of people...I was struggling at high school very early.” P01: “Just not having a stable life to even go to school… ” P08: “I wasn’t doing good in the last school I went to, so they put me into a boarding school.” |
| **Adaptive Survival and Emotional Self-Reliance** | Resource-driven survivalism | Personal / Structural | Material scarcity and exclusion “socialized” youth into street-efficacy that met immediate needs but normalized risk and surveillance. | Amid persistent scarcity and relational loss, youth developed two survival systems—resource-seeking and emotional withdrawal. These adaptive modes reflected agency under constraint: the capacity to sustain control by alternating between external hustling and internal self-protection. Together, they formed a self-reliant but isolating survival logic. | Scarcity socialization and autonomy-as-safety | P02, P05, P07 | P02: "I used to go to the corner stores and steal anything to eat." P05: "'Why is food expensive…and hygiene [products]... it's expensive in general." P07: "I probably just stole it [if I don't have the money]." |
|  | Protective autonomy and withdrawal | Personal / Interpersonal | To manage chronic instability and relational harm, youth turned inward by asserting hard boundaries and withdrawing to reduce vulnerability. |  | Protective withdrawal | P01, P07, P08 | P01: "It was just too much… I like to be alone…putting up boundaries with my mom" P07: "Temporary…exhausting…other people's energy…I like to be alone" P08: "Relationships slow me down…I shouldn't always depend on people." |
| **Cyclical Violence and Relational Fractures** | Re-enacting trauma through unsafe relationships | Interpersonal | Early trauma resurfaced in adolescence and early adulthood through coercive or violent relationships that mirrored earlier patterns of control and abuse. | Experiences of interpersonal and gendered violence mirrored earlier losses, reinforcing mistrust and emotional detachment. For many, care and harm coexisted, teaching that closeness often carried danger. Violence was not episodic but cyclical, woven through attachment and identity in ways that made protection and withdrawal equally necessary for survival. | Trauma reenactment and relational mistrust | P01, P05, P06, P08 | P01: "It got really bad… I had to go to the police… [my ex-partner] is a pedophile." P05: "[My partner] stabbed me in my lung." P08: "[M partner] insults me… hurtful things about the baby." |
|  | Gendered vulnerability and mistrust | Interpersonal / Intersectional | Participants negotiated safety and belonging within gendered and transphobic environments, developing cautious trust and protective withdrawal as adaptive responses to repeated harm. |  | Identity-based threat and adaptive boundary regulation | P01, P02 | P01: "The leaseholder’s boyfriend… very anti‑trans… I couldn’t say my pronouns… I was scared." P02: "Because I’m visibly queer person…My dad would not be as big of a problem if I was not [because] he didn’t want a gay daughter.” |
| **Navigating Systemic Contradictions** | Systems that help and hinder | Structural / Intersectional | Systems that offered aid simultaneously enforced surveillance and dependency, creating moral fatigue. | Early adulthood introduced ongoing negotiations between care and control. Youth encountered systems that offered resources while reinforcing surveillance and dependency. Across welfare, housing, and probation contexts, help and punishment coexisted, cultivating exhaustion and strategic disengagement. | Help–control paradox | P03, P06, P08 | P06: "You have to go through the system… juvenile, prison, homelessness just to get help." P08: "People act like they don’t want to help me… I go around different [housing] programs to ask." |
|  | Bureaucratic fatigue and inequities | Structural / Personal | Navigating complex housing and benefits systems caused confusion, burnout, and disillusionment with institutional support. |  | Bureaucratic fatigue and disillusionment | P03, P06 | P03: "They told my apartment that if I don’t respond… I’d lose my housing voucher." P06: "The system makes you lazy… relying on government help." |
| **Relational Repair and Conditional Support** | Healing through meaningful peer relationships | Interpersonal | Peers and chosen family restored trust, accountability, and emotional safety, countering earlier attachment loss. | Emerging adulthood was characterized by relational reconstitution. Youth began rebuilding belonging through peer networks, community programs, and transitional housing that offered both safety and surveillance. These environments fostered accountability and mutual aid but remained conditional, which is structured by time limits, eligibility, and rule compliance. | Horizontal care and reciprocal trust | P01, P02, P03, P04, P05, P07 | P01: "My friend really helped me… I got up on my feet with that." P02: "He was like a brother… teaching me life lessons." P03: "I met [my best friend] in 2016… we ended up being best friends and still are." |
|  | Shared communities as survival and solidarity networks | Community / Structural | Collective networks (punk scene, LGBTQ+ centers, transitional housing) offered identity affirmation and resource exchange. |  | Mutual aid and collective resilience | P01, P03, P04, P05, P08 | P01: "My housemates are super community-oriented…" P03: "The LGBT Center helped me get the voucher." P04: "I got really into the punk scene… always somebody there to help me." |
|  | Transitional and housing programs as scaffolds of stability | Community / Structural | Transitional housing programs offered structure and security but were time-limited and rule-bound. |  | Conditional stability and program dependency | P01, P06, P07, P08 | P01: "The [TYI] program did help me… I have somewhere to go at night." P07: "[Housing] programs helped me out… If it wasn’t for them, I’d probably still be [couch surfing]." P06: "[TYI] is a positive impact… preparing us to do it on our own." |
| **Stabilizing Through Structure and Regulation** | Structured independence and financial responsibility | Personal / Structural | Education, employment, and financial skills provided daily structure and a tangible sense of progress toward independence. | As youths entered young adulthood, they began consolidating stability through a layered integration of external structure and internal control. Education, employment, housing, and recovery practices provided daily routines that grounded independence, while relational boundaries and emotional regulation fostered safety and accountability. Stability emerged not as a single achievement but as an ongoing process of “regulated freedom,” where structure enabled autonomy and autonomy, in turn, reinforced structure. Through this interplay, participants transformed earlier cycles of instability into a sustained sense of agency and continuity. | Empowerment through structured mastery | P01, P03, P04, P06, P08 | P06: "I started back my education… College of XXX." P08: "I started the medical assistant program in October 2024." P08: "And I want to work so I can get my housing, so I don't know how that will turn out if I can't work." P03: “[The TYI program] was really helpful… with saving money...I did cosign a lease for my best friend… my credit score is really good.” |
|  | Boundary-setting and healthy ties | Personal / Interpersonal | Setting boundaries and cultivating selective relationships created safety and emotional regulation. |  | Relational regulation | P01, P02, P04, P08 | P01: "I can do my own thing… putting up boundaries with my mom." P02: “There’s people from placement that I still talk to… people who were supporting me through the bad times.” P08: "I told him we shouldn't be together only because I don't want to feel depressed while I'm having a baby and stuff like that. I feel like it's better that way...Relationships slow me down… I shouldn’t always depend on people." |
|  | Housing structure and stability as mutual stabilizers | Personal / Structural | Stable housing, freedom from institutional oversight, and personal regulation operated as mutually reinforcing mechanisms of safety and autonomy. |  | Developmental integration of structure and self-regulation | P01, P03, P04, P06, P07 | P01: “Structured housing… it’s healing… my nervous system is calming down… I’m six months clean.” P07: “Programs helped me out… If it wasn’t for them, I’d probably still be [couch surfing].” P06: "Well, the only thing that's saving me is that I'm under the affordable housing program." P06: "I was free [from probation]. I didn't have to check-in with no parole, got no probation officer, drug test, none of that." |
| **Reframing Pain into Purpose and Contribution** | Transformation, faith, and reflective practice | Personal / Interpersonal / Structural | Through reflection, therapy, and faith, youth reinterpreted trauma as evidence of strength and self-worth, transforming survival into personal growth. | After achieving basic stability, participants engaged in meaning reconstruction by reframing trauma into purpose through reflection, advocacy, and vision for the future. This shift from “being helped” to “helping others” marked a transformation from survival to contribution, embedding stability within identity and community. | Meaning reconstruction | P01, P02, P05, P06, P08 | P01: "I feel like lots of [my mental health challenges] have gone away, though, becoming an adult." P02: “I stopped stealing, stopped fighting… started turning to God… talking to my therapist… people look up to me.” P05: “My guardian told me to go to treatment… a sober community… get back on your feet.” P06: “[TYI] is doing the right thing… preparing us to do it ourselves.” |
|  | Mentoring, advocacy, and reciprocal care | Personal / Interpersonal | Helping peers and engaging in advocacy transformed adversity into leadership, reinforcing self-efficacy and belonging through service. |  | Reciprocal empowerment (using one's recovery to empower others) | P01, P02, P06 | P01: “People feel like I’m that bulletproof girl… I’m standing on my own two feet…People look up to me… I started to love my confidence.” P01: “Big corporations buying up multiple properties out here, hiking up prices, squeezing out money from everybody…nobody has a house anymore.” P06: "[The housing programs] prioritize single moms...they don't prioritize men." |
|  | Integration and future orientation | Personal / intersectional | Participants described a sense of peace, confidence, and forward movement as they integrated lessons from instability into a coherent adult identity. |  | Forward integration | P01, P02, P03, P04, P06, P08 | P01: “[Age] 25 is my first year of peace." P02: "And now I'm standing in my own two feet. I don't ask for handouts. I literally go getting myself. Nobody has to help me, nobody has to do anything. I'm a find a way for myself, because I'm an adult." P04: "And I was just doing what I could to be housed. And I worked like a few different jobs. I started playing music during that time. That was quite significant to the future going forward, and what I am doing now." P06: “[TYI] prepares us to put ourselves in the right direction.” P08: “I wish I had my own apartment… but I’m working toward it.” |
|  |  |  |  |  |  |  |  |
